# Primary pleural epithelioid angiosarcoma treated successfully with anti-PD-1 therapy: A rare case report

**DOI:** 10.1097/MD.0000000000027132

**Published:** 2021-09-03

**Authors:** Xia Wang, Jianping Wei, Zhimin Zeng, Jing Cai, Zhiqin Lu, Anwen Liu

**Affiliations:** a Department of Oncology, The Second Affiliated Hospital of Nanchang University, China; b Jiangxi Key Laboratory of Clinical Translational Cancer Research, China.

**Keywords:** anti-PD-1, case report, immunotherapy, pembrolizumab, pleural epithelioid angiosarcoma

## Abstract

**Rationale:**

Primary pleural angiosarcoma (PPA) is an extremely rare malignancy for which there is no consensus on treatment. The clinical course of PPA is usually quickly fatal, regardless of the treatment used.

**Patient concerns:**

We describe the rare case of a 52-year-old man who presented initially with hemoptysis and received emergency surgery for the primary.

**Diagnoses:**

He received a confirmed diagnosis of primary pleural angiosarcoma (PPA) by postoperative pathology and was subsequently treated with radiotherapy and chemotherapy, but had failed and was intolerant to chemotherapy.

**Interventions:**

The patient had 5% tumor PD-L1 positivity with 22C3 pharmDx and received pembrolizumab (200 mg every 21 days) for 13 cycles.

**Outcomes:**

The disease remained well controlled according to the RECIST 1.1. criteria. He is currently under observation and waiting to start the next cycle of immunotherapy.

**Lesson:**

Our case report suggests that the use of anti-PD-1 therapy does show efficacy in the treatment of PPA and may provide a viable treatment option for patients.

## 1 Introduction

Angiosarcoma (AS) is an uncommon malignant tumor of endothelial differentiation, accounting for only 1% to 2% of all soft tissue sarcomas.^[1,2]^ Primary pleural angiosarcoma (PPA), a highly malignant disease, is an extremely rare malignancy, with approximately 50 cases reported in the literature.^[3]^ The treatments for angiosarcoma include surgical excision, radiotherapy and chemotherapy. Nevertheless, the clinical course is usually rapidly fatal, regardless of the treatment applied. Approximately 75% of patients die from PPA within 7 months, and all die within 24 months.^[4]^ Recently, immunotherapy has become an important approach for treating patients with different cancers. Although these agents have been used in sarcoma therapy, their ability to treat PPA has not been reported. Here, we present the rare case of a patient with PPA treated with pembrolizumab who showed a well-established survival benefit.

## 2 Case report

**Figure 1:**
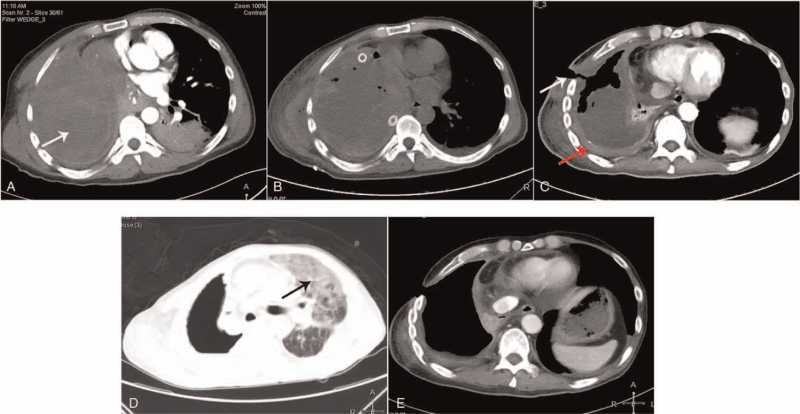
(A) CT images demonstrate a large mass (arrows) in the right thoracic cavity before emergency surgery (arrows). (B) CT images show pleural effusion and atelectasis of the right lung on the 12th postoperative day. (**C**) CT images show the chest sinus tract (white arrows) and thickening of pleural lesions (red arrows) after completing 2 cycles of nab-paclitaxel treatment. (D) CT images demonstrate grade 3 immune-associated pneumonia (arrows) after completion of the first cycle of pembrolizumab. (E) CT images demonstrate stable disease after completion of 7 cycles of pembrolizumab.

**Figure 2:**
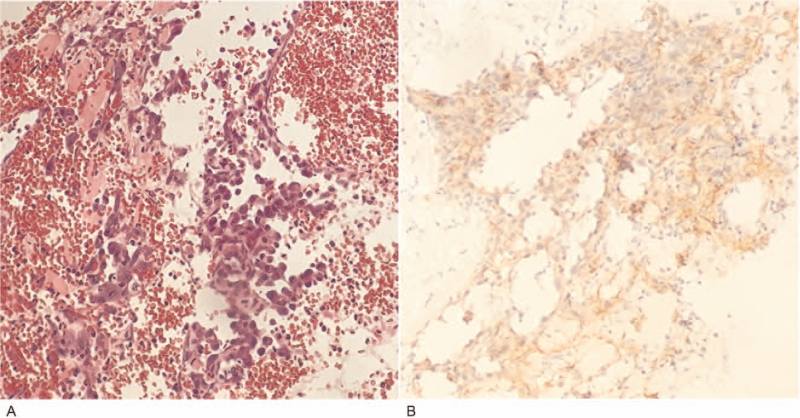
Photomicrographs of H&E staining (A) and immunohistochemistry results for PD-L1 (B) are shown. H&E staining showed angiosarcoma cells with hemorrhage and necrosis (200× magnification). Staining of the patient's tumor tissue revealed positive PD-L1 expression, with 5% of the tumor cells staining positive (400× magnification). H&E, hematoxylin-eosin. PD-L1 = programmed cell death-ligand 1.

A 52-year-old man presented with coughing and hemoptysis and was referred to our hospital. Chest computed tomography (CT) revealed a large mass in the right thoracic cavity complicated by internal hemorrhage, atelectasis of the right lung, massive infection and consolidation of the bilateral lungs (Fig. 1A). Because of repeated hemoptysis events, the patient underwent emergency thoracotomy and thoracic lesion non-radical excision. Pathological examination showed primary pleural epithelioid AS with hemorrhage and necrosis (Fig. 2A). The stage pTxN0M0, G3 were classified in accordance with the 8th edition of American Joint Committee on Cancer staging.

The patient exhibited hemoptysis throughout the postoperative course. Repeated CT revealed pleural effusion and atelectasis of the right lung on the 12th postoperative day (Fig. 1B). He underwent bronchial arteriography on the 18th postoperative day, but no bleeding artery was found. He immediately received radiotherapy to the right chest (30 Gy in 10 fractions) to constrain his hemorrhage. His symptoms were greatly relieved after radiotherapy. At 4 weeks after radiotherapy completion, he subsequently received 2 cycles of nab-paclitaxel. The clinical syndrome of effusion from the chest sinus tract and imaging studies (thickening of right pleura lesions) showed a progressive disease to treatment according to response evaluation criteria in solid tumors 1.1. (Fig. 1C), we determined that nab-paclitaxel monotherapy did not produce a good response. The patient was started on second-line chemotherapy with methotrexate, vincristine and propranolol in a 21-day cycle for 4 cycles.

**Figure 3:**
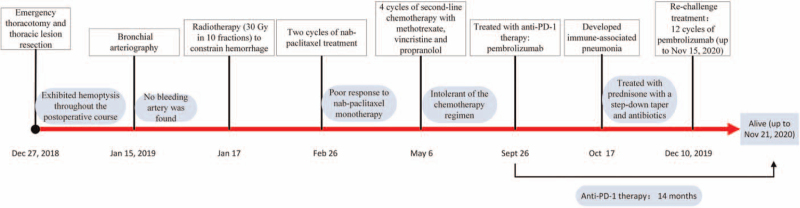
Timeline of the clinical history of the patient with primary pleural angiosarcoma.

Because of repeated degree III-IV neutropenia events, we had to apply new therapeutic modalities. The patient had 5% tumor programmed cell death-ligand 1 (PD-L1) positivity with 22C3 pharmDx (Fig. 2B). Based on this positive PD-L1 expression, treatment with pembrolizumab (200 mg every 21 days) was begun. After completion of the first cycle of pembrolizumab, he developed immune-associated pneumonia (Fig. 1D). Immunotherapy was suspended, and the patient was treated with prednisone with a step-down taper and antibiotics. After improvement in his lung condition, the patient subsequently received re-challenge treatment with 12 cycles of pembrolizumab. He had another chest CT study performed (Fig. 1E) that revealed stable disease after completion of 12 cycles of pembrolizumab. He is currently under observation and waiting to start the next cycle of immunotherapy. The timeline of the clinical history is summarized in Figure 3.

The patient signed informed consent forms for the publication of clinical data and images. This study was approved by the institutional ethics committee of the Second Affiliated Hospital of Nanchang University, Nanchang, China.

## 3 Discussion

PPA is an extremely rare malignancy. Given the rarity of PPA, its pathogenesis and etiology remain unclear and are still the object of much speculation. However, several exposure-related risk factors are known for this disease, including prior radiotherapy and a history of tuberculosis.^[5–8]^ Chronic inflammation and persistent physical stimulation are purported to result in PPA.^[9–11]^

The clinical symptoms of PPA are not specific and are commonly represented by chest pain, dyspnea, hemoptysis, pleural effusion and recurring hemothorax.^[12–15]^ Radiology provides multiparametric morphological and important help for the diagnosis of PPA, but the radiological signs are also not very specific. The diagnosis of PPA is strongly dependent on pathological findings. Biopsy or tumor resection performed by thoracotomy or by video-assisted thoracoscopic surgery were by far the most frequently applied and helpful diagnostic tool for PPA.^[16,17]^ Percutaneous lung/pleural biopsy was also supported by some cases.^[13,18,19]^ Pleural cytology had negative findings in all cases of PPA.^[16]^

Treatment modalities for PPA include surgery, radiotherapy and/or chemotherapy, and surgery followed by adjuvant radiotherapy is considered the optimal current treatment modality. One patient with a large solitary angiosarcoma was successfully treated with surgery and postoperative radiotherapy, remaining well and asymptomatic after 15 years of follow-up.^[12]^ Vascular embolization can be helpful in supporting hemostasis.^[20,21]^ There are no consensus criteria for chemotherapy regimens for PPA. Chemotherapy drugs included gemcitabine, docetaxel, paclitaxel, cisplatin, ifosfamide, doxorubicin, and/or albumin-bound paclitaxel.^[22]^ The role of chemotherapy may be limited. In addition, targeted medicines and immunotherapy have recently been studied as promising treatments for angiosarcomas.^[23–26]^ Tyrosine kinase inhibitors (TKIs) have been implemented in targeted therapy of angiosarcomas, especially sorafenib and pazopanib.^[23,25,26]^ In the previous studies, 4 patients were treated with targeted drugs: bevacizumab, pazopanib, and sorafenib.^[27,28]^

The evidence presented in this case suggests that the use of anti-PD-1 therapy does show efficacy in the treatment of PPA and may provide a viable treatment option for patients. Recently, a study described PD-L1 detection in 16/24 (66%) AS samples.^[29]^ We searched multiple databases but did not find a case report on PPA treated with anti-PD-1 therapy. Only one previous study reported a case of AS of the nose treated with pembrolizumab, in which the patient received pembrolizumab for 13 cycles, which resulted in marked shrinkage of his liver disease and no new facial lesions.^[30]^ Current treatment options for PPA are limited and poorly studied, making it necessary to consider treatment with TKI and checkpoint blockade therapies when appropriate. To the best of our knowledge, this is the first case report for a PPA patient treated with anti-PD-1 therapy and his immune-related toxicities were successfully controlled. Patients with PPA have limited treatment options, and it is important to consider treatment with anti-PD-1 agents when appropriate. However, more studies are needed to establish the efficacy and safety of agents targeting this immune checkpoint pathway in patients with PPA.

## Acknowledgments

We owe thanks to the patient and his family.

## Author contributions

**Funding acquisition:** Anwen Liu.

**Investigation**: Jianping Wei, Zhiqin Lu.

**Project administration:** Anwen Liu.

**Resources:** Jianping Wei.

**Software:** Jing Cai.

**Supervision:** Anwen Liu.

**Visualization:** Jianping Wei.

**Writing – original draft:** Xia Wang, Zhimin Zeng.

**Writing – review and editing:** Anwen Liu.
